# Energy Efficient Hybrid Routing Protocol Based on the Artificial Fish Swarm Algorithm and Ant Colony Optimisation for WSNs

**DOI:** 10.3390/s18103351

**Published:** 2018-10-08

**Authors:** Xinlu Li, Brian Keegan, Fredrick Mtenzi

**Affiliations:** 1 Department of Computer Science, Hefei University, Hefei 230601, China; 2 School of Computing, Dublin Institute of Technology, Dublin 8, Ireland; brian.x.keegan@dit.ie (B.K.); Fredrick.Mtenzi@dit.ie (F.M.)

**Keywords:** wireless sensor networks, hybrid routing protocol, ant colony optimisation, artificial fish swarm algorithm

## Abstract

Wireless Sensor Networks (WSNs) are a particular type of distributed self-managed network with limited energy supply and communication ability. The most significant challenge of a routing protocol is the energy consumption and the extension of the network lifetime. Many energy-efficient routing algorithms were inspired by the development of Ant Colony Optimisation (ACO). However, due to the inborn defects, ACO-based routing algorithms have a slow convergence behaviour and are prone to premature, stagnation phenomenon, which hinders further route discovery, especially in a large-scale network. This paper proposes a hybrid routing algorithm by combining the Artificial Fish Swarm Algorithm (AFSA) and ACO to address these issues. We utilise AFSA to perform the initial route discovery in order to find feasible routes quickly. In the route discovery algorithm, we present a hybrid algorithm by combining the crowd factor in AFSA and the pseudo-random route select strategy in ACO. Furthermore, this paper presents an improved pheromone update method by considering energy levels and path length. Simulation results demonstrate that the proposed algorithm avoids the routing algorithm falling into local optimisation and stagnation, whilst speeding up the routing convergence, which is more prominent in a large-scale network. Furthermore, simulation evaluation reports that the proposed algorithm exhibits a significant improvement in terms of network lifetime.

## 1. Introduction

Wireless Sensor Networks (WSNs) comprise a self-managed network system, which consist of numerous distributed sensor nodes. Generally, the purpose of a WSN is to monitor the conditions of an environment, such as temperature and humidity, by means of data collection [1]. In such networks, the sensor nodes usually have many limitations in terms of energy supply, computing and communication abilities. Hence, the core feature of the wireless sensor routing protocol is the energy efficiency; in other words, the energy-efficient routing protocol should extend the network lifetime and improve the quality of network communications. There are many current works and efforts that are ongoing for the development of the energy-efficient routing protocols in WSNs.

Hierarchical (clustering) technology is particularly promising and has received much attention in the research community. Multiple algorithms have been proposed to arrange the nodes in meaningful clusters for various applications, such as an adaptive clustering algorithm in [2] and Low-Energy Adaptive Clustering Hierarchy (LEACH) [3]. Researchers have utilised several schemes to select node clusters and to fuse the sensor data in each cluster and then relay the data towards the base station. These routing protocols combine energy-efficient cluster-based routing with application-specific data aggregation, achieve improved lifetime and achieve Quality of Service (QoS) requirements for WSNs [4,5].

Recently, many new routing protocols based on Ant Colony Optimisation (ACO) [6] have been developed. The basic idea behind the ACO algorithm for routing is that it imitates an ant colony’s foraging behaviour to define a nature-inspired meta-heuristic to discover the route to the destination. The first ACO-based routing protocol was presented for a wired network [7], then for ad hoc networks (MANETs) [8]. Zhang et al. [9] found that these protocols were not suitable for wireless sensor networks and subsequently presented a foundation framework for an ant-based WSN routing protocol. An Energy-Efficient Ant-Based Routing Algorithm (EEABR) [10] considers the energy factor of wireless sensor nodes on the basis of an ACO mechanism to extend network lifetime.

Furthermore, many species in nature show similar self-organisation and decentralised behaviour to that of an ant colony. Inspired by such behaviour, researchers presented many Swarm Intelligence (SI) optimisation algorithms [11]. A new SI algorithm, Artificial Fish Swarm Algorithm (AFSA), was proposed by Li et al. [12], inspired by the natural social behaviour of schools of fish. The basic idea of AFSA is to imitate fish behaviour such as *preying*, *swarming* and *following* with the local search of an individual fish capable of reaching the global optimum [13]. AFSA has many advantages including high convergence speed, flexibility, fault tolerance and high accuracy. AFSA has found wide-spread applications in complex optimisation domains, currently considered a major research topic, and offers an alternative to the more established evolutionary computation techniques that may be applied in many of the same domains [14].

The remaining sections of this paper are as follows: Section 2 provides a brief, but comprehensive overview of the state-of-the-art related works. In Section 3, we introduce our hybrid routing protocol based on AFSA and ACO in detail. In Section 4, a performance evaluation and discussion about the results are presented. Finally, the paper has been concluded in Section 5.

## 2. Related Work

### 2.1. Hierarchical Routing Algorithm

Hierarchical or cluster-based routing protocols are well-known techniques with special advantages related to scalability and efficient communication. In a hierarchical network architecture, all nodes are grouped into clusters. Every cluster has a cluster head, the election of which is based on different election algorithms. The cluster heads act as local coordinators to resolve channel scheduling, perform power measurement, maintain time division frame synchronisation and enhance the spatial reuse of time slots and codes [15].

Lin C R. et al. [2] considered a self-organising, multi-hop, mobile radio network for their proposed clustering algorithm. In the proposed hierarchical architecture, nodes are organised into non-overlapping clusters. The clusters are independently controlled and are dynamically reconfigured as nodes move. Simulation shows that this architecture provides an efficient, stable infrastructure for the integration of different types of traffic in a dynamic radio network.

An energy-aware clustering and resource management framework was presented by utilising the wireless powered communication (WPC) technique [4]. In the proposed framework, the network initially forms different clusters based on the Chinese restaurant process. Following this process, the cluster head is selected by considering the WPC paradigm. The performance of the proposed approach is evaluated via modelling and simulation and under various topologies and scenarios where its operational efficiency and effectiveness is demonstrated.

Tsiropoulou E. E. et al. [5] presented a distributed power control framework towards determining each node’s optimal transmission power in order to fulfil its QoS prerequisites. In the cluster formation process and the cluster head selection, the authors considered energy availability, interest and physical ties. The performance of the proposed approach is evaluated via modelling and simulation, and its improved performance is compared to other state-of-the-art approaches.

### 2.2. ACO-Based Routing

Ant Colony Optimisation (ACO) [6] is a swarm intelligence-based algorithm inspired by the search behaviour of an ant colony in nature. This search behaviour has the characteristics of being distributed, self-organised and having positive feedback. It has been observed in many of the existing research proposals that ACO is a widely-used technique to solve combinatorial optimisation applications due to these inherent features [16,17].

WSN applications require the sensor nodes to have a long network lifetime. Energy efficiency has been considered as a key issue in the design of routing protocols. In order to achieve this, it has become apparent that it is more important to route the data traffic such that the energy consumption is balanced among the nodes in proportion to their available energy instead of minimising the absolute consumed energy [18,19]. In recent years, several ACO-based energy-efficient routing algorithms were proposed to satisfy this inherent limitation of WSNs.

Sun, Y. et al. [20] improved the heuristic function in ACO and considered the node communication transmission distance and residual energy to find the optimal path of data transmission. The algorithm (IACO in [20]) ignores the effects of other energy levels (e.g., average energy, minimum energy) in the heuristic and pheromone update function, which could result in the energy dissipation of the entire network to run out of balance.

Ahmed et al. [21] proposed a self-optimising algorithm (SensorAnt) to optimise sensor node energy. The algorithm uses ACO to enhance the best route and achieve energy consumption balance. The method uses several routing metrics including the residual energy, the number of hops and the average energy of the route and network. The results of SensorAnt show performance improvements compared to EEABR [10] in terms of energy efficiency. However, the operation of SensorAnt depletes a significant amount of energy due to the effects of overhead. This energy depletion can be considered wastage and has a significant impact on the overall performance of the network.

Cheng et al. [22] proposed the Energy-Aware Ant Colony Algorithm (EAACA). The goal of EAACA is to create paths with low energy dissipation, balanced energy depletion and low transmission distance. Life Time Aware routing algorithm for Wireless Sensor Networks (LTAWSN) [23] utilised special parameters in its competency function for reducing the energy consumption of network nodes. A new pheromone update operator was designed to integrate energy consumption and hops into routing choice. However, during the route discovery and maintenance process of these two algorithms, several probe packets are sent to the destination node periodically, which results in a significant overhead and will increase the energy consumption.

The Multilevel Minimisation and Balancing for Energy Consumption (MMBEC) [24] achieves the objectives of energy hole avoidance and network lifespan prolonging for WSNs. MMBEC adopts a short-trip moving scheme in ACO, decreases algorithm complexity and improves convergence speed. Furthermore, the algorithm presents the concept of reference transmission distance, which helps to realise local energy consumption and consumption balancing. However, MMBEC assumes that nodes are uniformly deployed in the plane. If this condition is not met, a complex computation of the data is required to calculate the energy consumption of all sub-zones.

### 2.3. Hybrid SI-Based Routing

Recently, research has investigated hybrid combinations of the ACO-based routing algorithm and other optimisation algorithms, in order to solve some specific issues.

Fuzzy and Ant Colony Optimisation Based Combined MAC, Routing, and Unequal Clustering Cross-Layer Protocol for Wireless Sensor Networks (FAMCROW) [25] utilised fuzzy logic with residual energy, the number of neighbouring nodes and the quality of the communication link as input variables for cluster head selection. In doing so, the algorithm benefits from avoiding the hot spot problem. The ACO routing algorithm is adopted in inter-cluster multi-hop routing from cluster heads to the sink node.

Kaur et al. [26] presented a hybrid ACO and Particle Swarm Optimisation (PSO)-based energy-efficient clustering and tree-based routing protocol. In the protocol, hybrid ACO-PSO-based data aggregation comes in action to improve the inter-cluster data aggregation further.

The proposed routing technique [27] involves ACO and the Artificial Immune System (ANT-AIS), which aimed to balance energy exhaustion so as to maximise the network lifetime. In ANT-AIS, an ant colony algorithm is used for determining the optimum paths from sensor nodes to the sink. The artificial immune system is used for solving packet loop problems and controlling route direction.

R. Khoshkangini et al. [28] combined ACO with breadth-first search to discover the shortest path in order to improve data transmission with the least amount of energy consumption, as well as reducing the probability of data loss.

CB-RACOin [29] combines the ACO meta-heuristic with the computationally-inexpensive and distributed community detection technique Label Propagation (LP) [30], creates communities in WSNs and satisfies the balance of energy consumption by routing data inside communities through swarm intelligence.

Essentially, the design of the ACO-based routing algorithm lies in the probabilistic strategy of route discovery, the update of the pheromone trail and the heuristic function [31,32]. However, due to the inborn defects of the ACO-based routing protocol, the route discovery process is time consuming and cannot find a feasible route to the destination quickly. Due to the slow convergence behaviour, for a large-scale network routing optimisation problem, it is difficult to find an optimal path from a large number of chaotic paths in a short period of time.

Furthermore, the ACO-based routing protocol is prone to premature, stagnation phenomenon, which hinders further route discovery [33]. In ACO-based routing protocols, the basis of individual ant optimisation is pheromone concentration and heuristic value. However, at the beginning of the search, the pheromone concentration is uniform throughout the search space. Therefore, it cannot be guaranteed that the initial path selected by artificial ants is the one to reach the optimal path, which then in turn leads to stagnation [33,34]. After searching for a certain time, pheromone accumulates on the local optimal path, which leads to the convergence of the solution found by all artificial ant individuals. The pheromone accumulations and subsequent convergence prevents further searches for the global optimal solution and misses the opportunity to discover an optimal solution.

### 2.4. Artificial Fish Swarm Algorithm

Artificial Fish Swarm Algorithm (AFSA) [12,35] is an optimisation algorithm, which is based on swarm intelligence. In nature, the fish can discover a more nutritious area by individual search or following after other fish. The area with more fish is generally found to be most nutritious. AFSA has been successfully applied to many engineering design problems with superior performance compared to similar algorithms [36].

The basic behaviour of artificial fish is defined as follows:

1. Preying behaviour: This is a basic biological behaviour that tends toward food; generally the Artificial Fish (AF) perceives the concentration of food in water to determine the movement by vision or other sense and then chooses the tendency.
2. Swarming behaviour: The AF will assemble in groups naturally in the moving process, which is considered a living habit, in order to guarantee the existence of the colony and avoid dangers.
3. Follow behaviour: In the moving process of the artificial fish swarm, when a single AF or several ones find food, the neighbourhood partners will trail behind and reach the food quickly.

In AFSA, the AF can swim fast towards the direction with enough food by sensing and searching for food in the water. Preying behaviour, swarming and following enable AF confined in local extreme locations to move in the direction of a few AF tending to a global extreme value, which results in AF escaping from the local extreme values and thus accelerates the convergence speed of the algorithm [14,37].

Due to these inherent features, AFSA can achieve a satisfactory feasible solution quickly, although it lacks the ability to obtain accurate solutions. Furthermore, there is no need for the inspiration of the proposition’s prior knowledge, and it is not sensitive to the choice of initial parameters and can therefore be considered robust [38].

In this paper, after considering the operation of ACO-based routing, we take into account the inherent flaws, and we combine the characteristics of AFSA to propose a hybrid routing algorithm (named FSACO) for WSNs. The main contributions are summarised as follows:

- A novel AFSA-based initial route discovery algorithm is proposed. The AFSA algorithm achieves a comparatively better global optimisation performance in the early stage and can obtain higher quality routes quickly [12,14]. We utilise these feasible routes as a heuristic factor for the ACO routing algorithm in our hybrid routing protocol, which can improve the power efficiency of the entire hybrid swarm intelligent routing protocol.
- We propose a hybrid route discovery algorithm, which combines the crowd factor in AFSA and the pseudo-random proportional route select model in ACO-based routing algorithms. The introduction of the crowd factor can effectively prevent the routing algorithm from falling into a local optimum and find the optimal path finally.
- Propose an improved pheromone trail update method based on energy levels and path length to prolong the network lifetime.

## 3. Protocol Specification

### 3.1. Network Model

In this paper, we employ an elementary model for describing WSNs as undirected and weighted graphs. As such, let G=(V,E,W) be a simple weighted graph, in which *V* is the set of nodes denoted by positive integer numbers from one to $\left|V\right|$, representing the sensor nodes and the sink. The set *E* is composed of links, here expressed by $e_{i,j}$, where i,j∈V. For each $e_{i,j}\in E$, there is a $w_{i,j}\in W$ attached, which denotes the energy-related function.

Under this network model, a source node periodically senses and collects the data from the surroundings, then sends the data to the next hop until the data reach the sink node. The goal is to find an energy-efficient route such that the energy consumption is balanced and the lifetime of the network prolonged. Moreover, we assume that:

1. All nodes are isomorphic;
2. Links are symmetric. If the target’s transmitted power is known, nodes can calculate the approximate distance of senders according to the Received Signal Strength Indication (RSSI);
3. Depending on the recipient’s distance, the node can adjust its transmit power.
4. We use a first radio model as described in [3].

Based on these assumptions, the transmitter power level can be adjusted to use the minimum energy required to reach the intended next hop receiver, then the energy consumption rate per unit information transmission depends on the choice of the next hope node, i.e., the routing decision.

### 3.2. Protocol Overview

Informally, our hybrid routing protocol based on AFSA and the ACO routing algorithm, named FSACO, works as follows:

1. First, AFSA is initialised for each AF to perform preying, swarming and following behaviours from the source node to the sink node.
2. After several iterations, a selection of satisfactory routes to the sink node is obtained through the AFSA algorithm.
3. Initialise the pheromone information of the ACO routing algorithm using the satisfactory routes obtained by AFSA.
4. At some variable interval, a Forward Ant (FANT) is launched from the source node toward the sink node.
5. Each FANT chooses the next node according to a probabilistic state transition rule integrated with the crowd factor in AFSA.
6. Once FANT reaches the sink node, a Backward Ant (BANT) is created, which moves back along the route that the FANT had previously traversed.
7. During the BANT travel, it will modify the amount of pheromone on the path by applying the pheromone updating rule.
8. Once a BANT arrives at the source node, the next iteration will begin after some interval.

### 3.3. Initial Route Establishment

In ACO-based routing algorithms, the pheromone is initialised to a small uniform value for the start of the route discovery. The probability distributions of route selection will not be updated until the first artificial ant arrives at the sink and returns back. Hence, the distributions do not guide the artificial ants, which end up constructing (and reinforcing) paths of very bad quality. As discussed in Section 2, AFSA demonstrates exceptionable global search abilities in the early stage of route discovery and can obtain high quality satisfactory routes quickly. Furthermore, AFSA is not sensitive to the initial parameter values. Therefore, we use the positive qualities of AFSA to apply the preferred routes obtained by AFSA as the initial pheromone value of the following ACO routing algorithm.

Initialise the state vector of the artificial fish swarm attached to each sensor node as $X=(x_{1},x_{2},\ldots ,x_{n})$, n∈N; *N* represents the number of sensor nodes; $x_{i}\left(i=1,2,\ldots ,n\right)$ denotes the residual energy of current node. The sensory distance and the crowd factor are represented by visual and δ(0<δ<1). In our network model, visual represents the communication radius of sensor node, which means the nodes in visual of node *i* are the neighbour nodes of node *i*. $N_{i}$ denotes the set of nodes within the visual of node *i*. Crowd factor δ represents the congestion degree around a certain sensor node, which is used to avoid over crowding or collision with neighbouring regions. We also define a fitness function $Y=f(x_{i})$, which stands for the average energy within the communication radius of sensor node *i*.

(1) $$Y=f\left(x_{i}\right)=\frac{1}{N}\sum_{j\in N_{i}}x_{j}$$

All the AF search for the optimal route toward the sink node using three distinct properties, namely preying, swarming and following.

#### 3.3.1. Preying Behaviour

Preying is the basic biological behaviour adopted by AF looking for food. Generally, an AF perceives the region with more residual energy by vision or other sense and moves quickly towards this sensed region. Suppose the current state of an AF is $x_{i}$, i.e., residual energy of the current sensor node; it selects the next node randomly within its visual distance (sensory distance), such that:

(2) $$x_{j}=x_{i}+rand\left(0,1\right)\times visual$$

where $x_{j}$ is the current state and $x_{i}$ is the previous state. If the fitness function $f\left(x_{j}\right)>f\left(x_{i}\right)$, i.e., there is higher energy density around node *j*, the AF in node *i* goes forward to node *j*; if not, select a node $x_{j}$ randomly again, and judge whether it satisfies the forward requirement or not. If the forward requirement cannot be satisfied after *try_number*, the AF would move a step randomly, and this can help the AF flee from the local extreme field.

#### 3.3.2. Swarming Behaviour

Let the current state of AF be $x_{i}$ and $\left|N_{i}\right|$ be the number of its neighbour nodes within the visual distance. If $\left|N_{i}\right|\neq 0$, the maximum fitness value in neighbour nodes is defined:

(3) $$Y_{max}=\max_{j\in N_{i}}f\left(x_{j}\right)$$

where $N_{i}$ represents the set of neighbour nodes of node *i*. If $\frac{Y_{max}}{\left|N_{i}\right|}>\delta \times Y_{i}$, that means the node with maximum fitness is not very crowded. If $Y_{max}>Y_{i}$, the AF moves towards the node *j*, otherwise, the AF executes the preying behaviour according to Equation (2). Here, the crowd factor δ limits the scale of the artificial fish swarm, causing more AF to cluster at the area with more average residual energy, which ensures that AF move to an optimum in a wide field.

#### 3.3.3. Following Behaviour

Following behaviour accelerates AF moving to better states and at the same time accelerates AF moving to the global extreme value from the local extreme values. When an AF finds food, neighbouring AF will trail behind and reach the food.

Suppose the $x_{s}$ represents the sink node within $x_{i}$ visual distance and $Y_{s}=f\left(x_{s}\right)$ is the fitness value of the sink node. If $\frac{Y_{s}}{\left|N_{i}\right|}>\delta \times Y_{i}$ and $Y_{s}>Y_{i}$, then the AF swims toward the sink node, otherwise, the preying behaviour is executed.

After several iterations, AFSA will find satisfactory feasible routes to the sink node. Furthermore, the AF leave positive feedback information in each link, which will be used in the ACO-based route discovery process described in Section 3.4.

### 3.4. Hybrid Route Discovery

In our proposed hybrid routing algorithm based on AFSA and ACO, we use the heuristic information from AFSA as an initial pheromone value in the early stage in the ACO route discovery process, which is expected to avoid chaos and falling into a local optimum.

We introduce a novel probabilistic route discovery scheme, which takes its inspiration from a state transition rule in the Ant Colony System (ACS) [6]. A sensor node *i* releases a forward ant toward the sink node at some interval and will select the next node *j* to move to by applying the rule given by Equation (4):

(4) $$j=\left\{\begin{array}{cc} \underset{r\in N_{i}^{k}}{arg max}\;\left\{\tau_{ir}^{\alpha }\cdot \eta_{ir}^{\beta }\right\} & ,q\leq q_{0}\left(exploitation\right)\\ p_{ij} & ,q>q_{0}\left(exploration\right) \end{array}\right.$$

where *q* is a random number uniformly distributed in [0,1], $q_{0}$ is a control parameter $\left(0\leq q_{0}\leq 1\right)$ of route exploitation and exploration and $p_{ij}$ is a random variable selected according to the probability distribution given in Equation (5):

(5) $$p_{ij}=\left\{\begin{array}{cc} \frac{\tau_{ij}^{\alpha }\cdot \eta_{ij}^{\beta }}{\sum_{r\in N_{i}}\tau_{ir}^{\alpha }\cdot \eta_{ir}^{\beta }} & ,\forall j\in N_{i}\;and\;j\notin M\\ 0 & ,otherwise \end{array}\right.$$

where $\tau_{ij}$ and $\eta_{ij}$ refer to the global pheromone trail and the local heuristic desirability of link (i,j), respectively. α and β are two parameters that control the relative importance of pheromone trail and heuristic value. $N_{i}$ is the set of neighbours of node *i*. *M* is the tabu list, which stores the visited node and carried by the forward ant.

According to this method, a sensor node will select the optimal neighbour node (exploitation) or a stochastic one (exploration). Here, in Equation (4), exploitation implies that the forward ant has the ability to exploit prior and accumulated knowledge, while exploration means that the artificial ant pays more attention to exploring new paths toward the sink node. This method will reinforce the positive effect of an artificial ant learning process and then increase the convergence speed for the route discovery in order to achieve a better path in the early route discovery phase.

However, at the later stage of the algorithm, the concentration of pheromone in a certain path continuously increases. The ACO routing protocol has a higher probability of being trapped in the optimal layout according to Equations (4) and (5) [39]. Here, we introduce the crowd factor $\delta_{ij}$ in AFSA to limit the scale of pheromone increment.

(6) $$\delta_{ij}=2\tau_{ij}/\sum_{i\neq j}\tau_{ij}$$

where $\delta_{ij}$ and $\tau_{ij}$ refer to the pheromone value and crowd value of link (i,j), respectively. At the same time, we use δ(t) to indicate the crowd threshold at time *t*. If $\delta_{ij}<\delta \left(t\right)$, which means the current link is less crowded, the artificial ant will follow that link toward the next sensor node, otherwise, the artificial ant has to re-select the next hop according to Equation (5). The crowd threshold value is a function of time *t*.

(7) $$\delta \left(t\right)=1-e^{-\lambda t},\;\;\;\;t\geq 0\;\;and\;\;0<\lambda \leq 1$$

where λ is constant and *t* is the route discovery iteration times. In this way, the crowd threshold value will change with the route discovery times. The larger *t* is, the larger δ(t) will be.

In the initial phase of the algorithm, the crowd factor $\delta_{ij}$ and the crowd threshold δ(t) are both small; thus, the crowd factor does not restrict the route selection process. As the iterative process continues, the concentration of pheromone in some links increases significantly, and the crowd threshold also gradually increases. In this case, the crowd factor will limit the artificial ant influx into the high pheromone link. The introduction of a crowd factor controls the number of artificial ants in the overcrowded path. This hybrid algorithm can effectively prevent the artificial ants from prematurely aggregating on a path with high pheromone to cause the premature routing.

At the end of route discovery, the impact of the crowd factor becomes insignificant according to Equations (6) and (7). The hybrid route discovery algorithm based on AFSA and ACO degenerates into the ACO routing algorithm whose route selection depends on Equations (4) and (5).

In general, in the initial stage, this hybrid routing algorithm avoids overcrowding of the sub-optimal route, while in the later stage, it can ensure that the routing algorithm can quickly converge to the optimal route.

### 3.5. Global Pheromone Update Strategy

The global pheromone update procedure will be applied when the FANT arrives at the sink node. The global pheromone includes information about a route obtained by long-term learning from a FANT. After arriving at the sink node, the FANT will be converted into a BANT. The BANT inherits all route statistic information from the FANT, including path length, energy levels and the visited node list along that path. Meanwhile, the sink node calculates the amount of pheromone value attached to this path according to the route statistics. We calculate Δτ in the following manner:

(8) $$\Delta \tau =\frac{E_{min}\cdot E_{avg}}{e^{E_{init}}\cdot F_{ant}}$$

$E_{min},E_{avg}$ and $E_{init}$ represent the minimum energy, average energy and initial energy in the current discovered path respectively, and $F_{ant}$ denotes the length of the path. The BANT carries Δτ at the start of its journey following the reverse path. Each node in the path updates the global pheromone trail (i.e., routing table) according to Equation (9).

(9) $$\tau_{ij}=\left(1-\rho \right)\cdot \tau_{ij}+\rho \cdot \Delta \tau_{ij}$$

where 0<ρ<1 is the pheromone evaporation coefficient and (1−ρ) is the pheromone residue factor, and $\Delta \tau_{ij}$ is given by:

(10) $$\Delta \tau_{ij}=\left\{\begin{array}{cc} \xi \cdot \frac{E_{j}}{E_{inti}\cdot B_{ant}}\cdot \Delta \tau & ,i,j\in M\\ 0 & ,i,j\notin M \end{array}\right.$$

where 0<ξ<1 is a control coefficient, which normalises $\Delta \tau_{ij}$ to the interval (0,1). $E_{j}$ is the residual energy of node *j*, from which a BANT has come. $B_{ant}$ denotes the path length from the sink node to the current node. The pheromone value is the function of both energy levels and the length of the path. As a result:

1. The shorter path (less hops) will get a larger pheromone increment.
2. When the minimum energy value in this path is larger, the pheromone increment is also larger, which avoids more data traffic routed through this path.
3. The average energy of a relatively high path will get more attention, and therefore will attract more data flow.
4. The closer the node is, the more pheromones are obtained.

## 4. Performance Analysis

To assess the performance of the proposed FSACO protocol, we developed a study under a simulated environment on NS-2 (Version 2.35). We obtained the results in terms of different performances from simulating FSACO in comparison with EEABR [10], SensorAnt [21] and IACO [20] mentioned in Section 2.

### 4.1. Simulation Model and Parameters

The parameters in our simulation are shown in Table 1. Since the parameters in ACO greatly influenced the performance of the algorithms, we adopted the values of 1, 5 and 0.5 for α, β and ρ by default in accordance with the best practice from [40]. Furthermore, we set the constant λ in Equation (7) as 0.1 and control coefficient ξ in Equation (10) as 0.9 for the simulation. Furthermore, the parameter try_number in the AFSA-based initial route discovery algorithm has an impact on the control overhead performance of the routing algorithm. A larger try_number allows an AF individual to more fully explore a new node in its neighbourhood, as compared with a small try_number. However, a larger try_number consumes more control packets. Here, we adopted the value 10 for try_number by default according to the [41].

For the series of simulations carried out, we use the network model as described in Section 3.1. A static network scenario was adopted to cover most, if not all, of the types of scenarios the algorithms might face, by varying the node density (number of nodes). The node density was varied in the range [100,500] in steps of 50.

### 4.2. Evaluation Metric

From several results obtained from our simulation experiments, we made use of the following performance metrics for evaluation purposes.

Energy consumption: The energy consumption is the sum of used energy of all the sensor nodes in the network during the period of the experiment in Joules (J). We estimate the node energy consumption considering the energy model proposed in [3].

Energy efficiency: This denotes the ratio of the total packets delivered at the sink node to the total energy consumed by the sensor nodes (Kbits/J).

Energy standard deviation. This gives the average variance between energy consumed on all nodes. It describes the energy consumption distribution of the networks. A low standard deviation indicates that the data points tend to be close to the expected value of the set, while a high standard deviation indicates that the data point is spread out over a wider range of values. The motivation behind this definition is that the algorithms should result in a low energy consumption standard deviation, which implies an even consumption distribution and good load-balance.

Network lifetime prediction. This is defined as the difference of the total initial energy of the network and the summation of the average used energy of nodes and the standard deviation of their energy levels.

(11) $$Lifetime\;Prediction=E-(\bar{\mu }+\sigma )$$

where *E* is the total initial energy, $\bar{\mu }$ is the average used energy, $\sigma^{2}={\left(\mu_{i}-\bar{\mu }\right)}^{2}/N$, *N* is the total number of nodes in network and $\mu_{i}$ denotes the residual energy of node *i*. The basic motivation behind this definition is that an algorithm should try to maximise average remaining energy levels of nodes, but with a minimal standard deviation.

Route setup time: This represents the time spent by a protocol to discover routes from the source node to the sink node. It is used to measure the time that the first effective route is discovered. We design a series of dynamic networks of different sizes to analyse this metric and evaluate the performance of the route discovery scheme and local heuristic update strategy in our proposed routing algorithm.

Convergence time: We define the convergence time as the optimised route setup time. It is complex and non-trivial to detect if the optimal route is established. Nonetheless, for an ACO algorithm used in simulated routing algorithms, convergence has been proven [42], which means these routing algorithms will discover an optimal route in a finite time. Here, we arrive at the convergence time by measuring the interval between the detection of the stable optimal route and route discovery initialisation.

Throughput: The number of messages per second received at thedestination. The throughput of the network is the sum of the throughput from all destinations.

### 4.3. Simulation Results

All results are summarised in Table 2, which represents a static network with 100 sensor nodes after running 300 s of simulation time. In such a scenario, there was one sink node, while the remaining nodes acted as sensor nodes to collect sensing data.

#### 4.3.1. Route Setup Time

**Figure 1:**
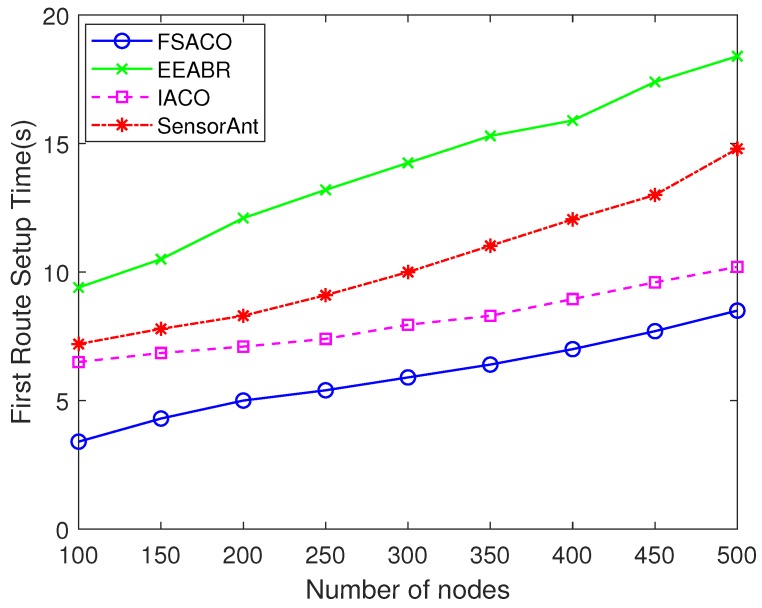
Impact of network scale on first route setup time.

It can be seen from Table 2 that FSACO could find the first effective route to the sink node faster than EEABR, IACO and SensorAnt under such a static network scenario. However, the difference of route setup time in Table 2 between FSACO and the other simulated routing algorithms was minuscule. The performance was related to the node density (number of sensor nodes) in the network. Figure 1 reports the impact of node density on the first route setup time.

As shown in Figure 1, in a 500-sensor-node network, FSACO found the first effective path 53.8%, 16.7% and 42.6% faster than EEABR, IACO and SensorAnt, respectively. In FSACO, we introduced the AFSA-based initial route discovery algorithm, which achieved improved global optimisation ability in the early stage and could obtain higher quality routes quickly. Hence, we can see that FSACO could find the first feasible route more quickly than the other tested protocols.

#### 4.3.2. Convergence Time

Table 2 demonstrates that EEABR, IACO and SensorAnt generally obtain stable optimal routes after about 130 seconds for a 100-sensor node network scenario. According to these findings, FSACO had 28.8%, 19.7% and 28.4% faster convergence speed than EEABR, IACO and SensorAnt, respectively. As mentioned in Section 2, the ACO-based route discovery algorithm used in EEABR, IACO and SensorAnt is prone to premature, stagnation phenomenon, whilst further route discovery is hindered, which results in sub-optimal routes. In FSACO, we introduced the dynamic crowd factor (Equations (6) and (7)) to help the number of artificial ants in the overcrowded link be controlled. This hybrid scheme could prevent the ants from prematurely aggregating on a path with high pheromone to cause premature routing. Ultimately, FSACO would accelerate the convergence of a stable optimal route, as reported in Table 2.

**Figure 2:**
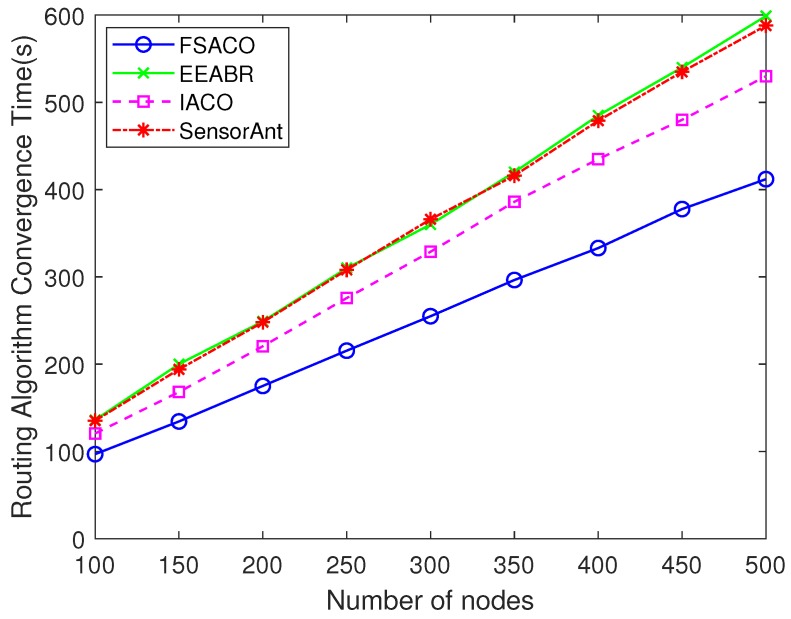
Impact of network scale on routing algorithm convergence.

Intuitively, as the network scale increased, routing protocols took more time to find the optimal route, which means that the convergence time would increase as a consequence. This has been proven in other applications of ant colony optimisation algorithms [16,41]. In Figure 2, we can observe that with the increase of network sensor nodes, FSACO, EEABR, IACO and SensorAnt required more time to find the stable optimal route; moreover, FSACO always achieved the fastest convergence speed in different network densities. Furthermore, with the increase of network scale, this difference became more apparent. In a 500-sensor-node network, the convergence performance of FSACO was 31.2%, 22.3% and 29.8% higher than EEABR, IACO and SensorAnt, respectively.

**Figure 3:**
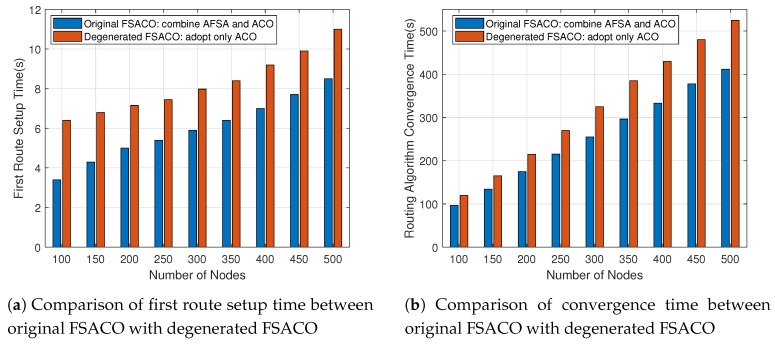
Analysis of the impact of Artificial Fish Swarm Algorithm (AFSA) optimisation on the FSACO routing algorithm’s performance in different network scales.

In order to assess the performance impact of the AFSA optimisation on the FSACO routing algorithm, we divided FSACO into two variants and analysed the different performance in terms of the first route setup time and routing convergence time. The results are reported in Figure 3. Here, original FSACO adopted the hybrid routing algorithm combined with AFSA and ACO, while degenerated FSACO only employed the pseudo random proportional rule in Section 3.4 and global pheromone trail update strategy in Section 3.5. Figure 3a demonstrates that the original FSACO had a shorter first route setup time than degenerated FSACO under the different node densities. This result proved that AFSA-based initial route discovery could help the routing algorithm find the feasible route to the sink node quickly. Figure 3b indicates that original FSACO sped up the routing convergence time over degenerated FSACO due to the hybrid route discovery algorithm. The reason is that the crowd factor in AFSA could effectively prevent the routing algorithm from falling into a local optimum. Furthermore, this effect was more pronounced in large-scale networks.

#### 4.3.3. Energy Consumption

**Figure 4:**
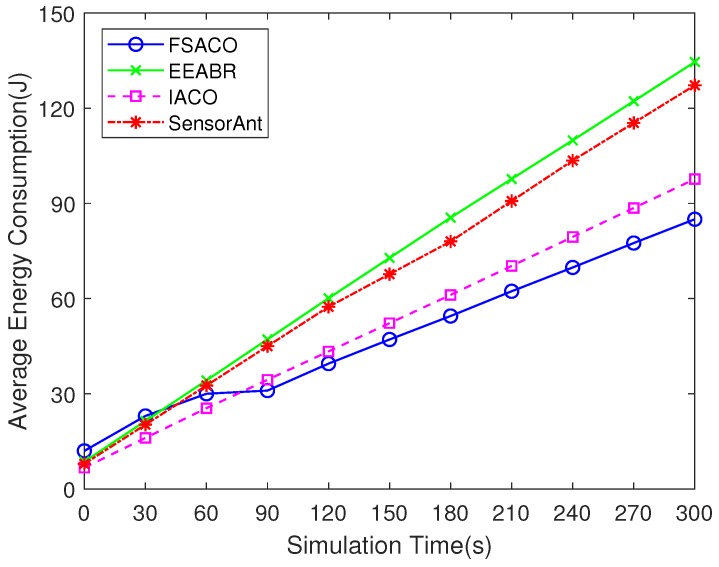
Comparison of energy consumption of FSACO, EEABR, IACO and SensorAnt under a static network scenario with 100 sensor nodes.

Figure 4 illustrates the trend of average energy consumption in sampling time. For all simulated routing protocols, it is clear that sensor nodes consumed more energy as the simulation progressed. FSACO indicated improved performance compared with EEABR, IACO and SensorAnt, by 36.8%, 13% and 33.2% improvement at the end of simulation, respectively. It is worth noting that FSACO consumed more energy than other routing algorithms in its initial stage. This was due to the artificial fish flooding in the AFSA-based initial route discovery algorithm, described in Section 3.3, which led to extra energy consumption.

#### 4.3.4. Energy Standard Deviation

We used the energy standard deviation to evaluate the balanced consumption of energy. More specifically, an energy-efficient routing algorithm should maximise the average remaining energy levels of nodes, but with a small standard deviation to prolong the network lifetime.

**Figure 5:**
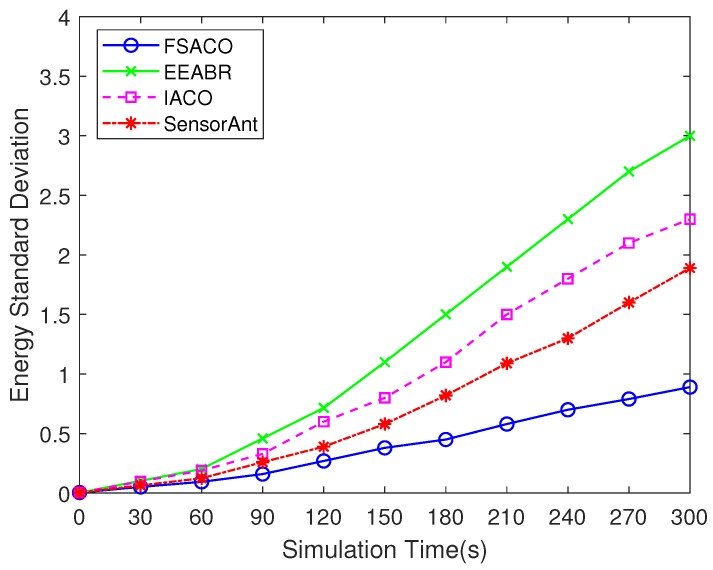
Comparison of the energy standard deviation of FSACO, EEABR, IACO and SensorAnt under a static network scenario with 100 sensor nodes.

In Figure 5, FSACO demonstrates the significant performance improvement by 70.3%, 61.3% and 52.9% over EEABR, IACO and SensorAnt in terms of energy standard deviation, respectively. This result was due to the introduction of the crowd factor in the route discovery phase, which avoided the premature stagnation phenomenon in EEABR, IACO and SensorAnt. As a result, the proposed hybrid routing algorithm in Section 3.4 helped to balance the consumption of energy in the network and avoid the rapid deterioration of the optimal path.

#### 4.3.5. Energy Efficiency

**Figure 6:**
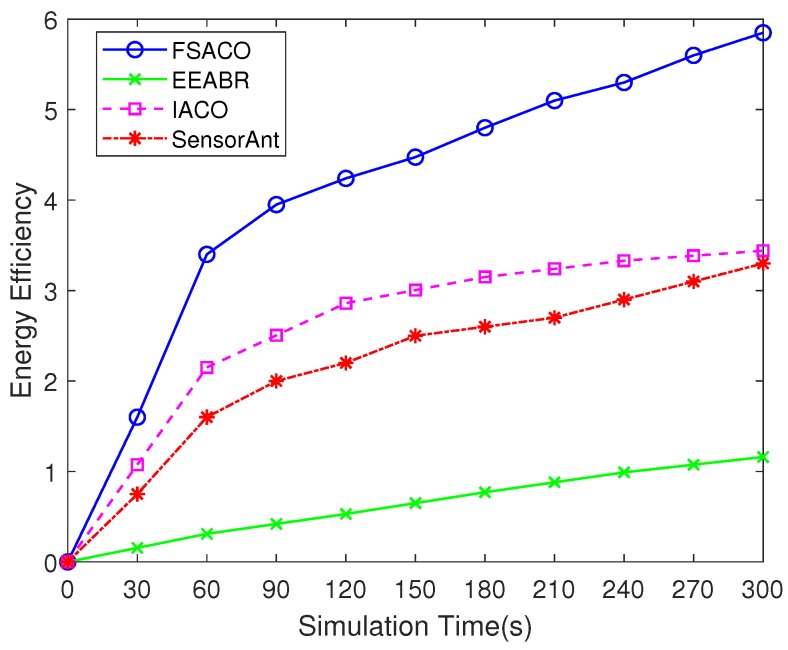
Comparison of the energy efficiency of FSACO, EEABR, IACO and SensorAnt under a static network scenario with 100 sensor nodes.

Figure 6 indicates the energy efficiency of routing protocols. The results indicated that the energy efficiency of FSACO was 5.04-times better than EEABR, 1.7-times better than IACO and 1.8-times better than SensorAnt. This means FSACO could deliver more sensing data to the sink node, while requiring equivalent energy consumption. The reason is that FSACO could find the initial effective route (i.e., feasible route) to the sink node quickly and the convergence time was less than that of other routing algorithms. This means FSACO could establish a stable optimal route quicker than EEABR, IACO and SensorAnt; hence, FSACO could deliver more sensing data to the sink node during in the same sampling time.

#### 4.3.6. Network Lifetime Prediction

**Figure 7:**
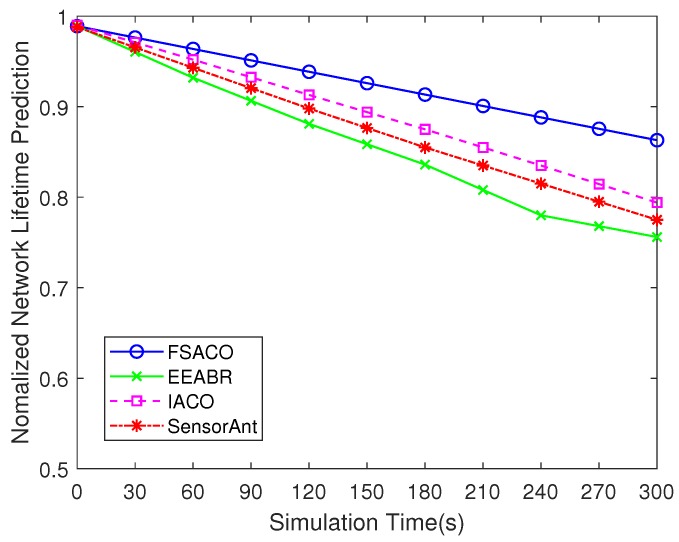
Comparison of network lifetime predication of FSACO, EEABR, IACO and SensorAnt under a static network scenario with 100 sensor nodes.

In Section 4.2, lifetime prediction is the function of initial energy, average energy consumption and energy standard deviation. Figure 7 reports a comparison of normalised network lifetime prediction of the network running FSACO, EEABR, IACO and SensorAnt. Due to a lower average energy consumed in FSACO (Figure 4) and a significantly improved performance in the standard deviation (Figure 5), hence, FSACO had a higher expectation in terms of network lifetime. The result obtained from Figure 7 indicated that FSACO could provide longer network lifetime prediction than that of EEABR, IACO and SensorAnt, respectively.

#### 4.3.7. Network Throughput

**Figure 8:**
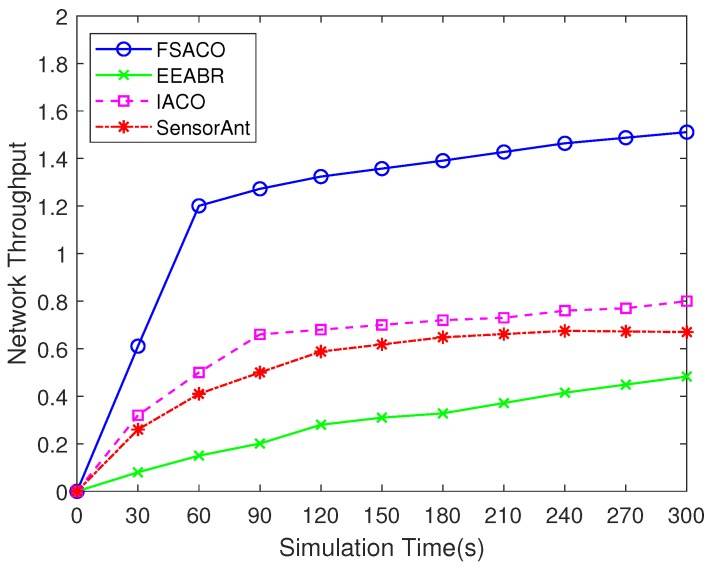
Comparison of network throughput of FSACO, EEABR, IACO and SensorAnt under a static network scenario with 100 sensor nodes.

Figure 8 demonstrates the impact of the proposed algorithms on average network throughput in a static network. The results obtained show that FSACO had better performance when compared with EEABR, IACO and SensorAnt. In the initial stage of route discovery, due to the probabilistic route discovery, the throughput was low. One may notice that the performance tended to be stable after several iterations. For example, FSACO would take about 60 s to reach stability. Moreover, the route establishing time of these algorithms was also very different. The results in the first route setup time and routing algorithm convergence time (Table 2) indicated this phenomenon.

In our proposed hybrid FSACO routing algorithm, the data transmission preferred the route with the shorter path as described in Section 3.5. Hence, the source node could effectively transmit more sensing data to the sink node.

## 5. Conclusions

ACO-based routing algorithms described in Section 2 have some common issues due to their intrinsic characteristics. First, due to the inborn defects of the ACO-based routing protocol, the route discovery process is time consuming and cannot find a feasible route to the destination quickly. Second, ACO-based routing protocols are prone to premature, stagnation phenomenon, which hinders their further route discovery.

This paper proposes a hybrid routing algorithm combining AFSA and ACO to solve these issues. We present an AFSA-based initial route discovery to obtain effective feasible routes towards the sink node quickly. The simulation results demonstrate that the algorithm can speed up the time of first route establishment, as well as route discovery convergence time. This performance improvement is more prominent in large-scale networks. Furthermore, to avoid the stagnation phenomenon in ACO-based routing algorithms, FSACO combines the crowd factor in AFSA and the pseudo-random proportional route select model in ACO to propose a hybrid route discovery algorithm. According to the simulations, the dynamic crowd factor can effectively prevent the routing algorithm from falling into a local optimum and find the stable optimal path finally. Furthermore, FSACO presented an improved pheromone update strategy by considering both the energy levels and path length as key metrics when updating the local heuristic value and pheromone trail. The simulation results reported that FSACO showed a significant improvement over EEABR, IACO and SensorAnt in terms of energy efficiency and network lifetime.

All of our results were obtained from simulation evaluation. However, the modelling of network behaviours in a simulation does not always reflect real-world scenarios. More accurate evaluation results can be gained through the implementation in a physical environment. We plan to implement the proposed algorithm on a physical test bed to study the impact of varying conditions on the performance of these algorithms.

## Figures and Tables

**Table 1:** Simulation parameters.

Parameter	Value	Parameter	Value
Dimension of topology	1000 $m^{2}$	Number of nodes	*100–500*
Nodes placement	*Random*	Mobility	*Static*
Traffic	Constant Bit Rate (*CBR*)	Simulation time	*300 s*
MAC layer	*IEEE 802.11*	Initial energy	*1000 J*

**Table 2:** Overall performance comparison between Energy-Efficient Ant-Based Routing Algorithm (EEABR), IACO, SensorAnt and FSACO.

Metric	EEABR	IACO	SensorAnt	FSACO
Route Setup Time	9.4	6.5	7.2	3.4
Convergence Time	136	120.5	135.2	96.8
Energy Consumption (J)	134.55	97.65	127.22	85
Energy Efficiency	1.16	3.44	3.3	5.85
Energy Standard Deviation	3	2.3	1.89	0.89
Lifetime Prediction	0.76	0.79	0.775	0.86
Network Throughput	0.48	0.8	0.67	1.511
